# A Review of Weight Loss With Glucagon-Like Peptide 1 Agonist Therapy: Is There a Role for Its Preoperative Use in Gynecology?

**DOI:** 10.7759/cureus.90764

**Published:** 2025-08-22

**Authors:** Annika Sinha, Evan R Myers, Anthony G Visco

**Affiliations:** 1 Urogynecology and Reconstructive Pelvic Surgery, Duke University Health System, Durham, USA; 2 Duke-Margolis Institute for Health Policy, Duke University Health System, Durham, USA

**Keywords:** glucagon-like peptide-1 agonist, hysterectomy, optimization, surgery, women’s health

## Abstract

Glucagon-like peptide 1 (GLP-1) agonist use for weight loss has skyrocketed. Dramatic weight loss with GLP-1 agonist use is frequently portrayed on social media. Contrastingly, such extensive weight loss has not been reported in published randomized controlled trials on weight loss after GLP-1 agonist use. However, these medications have been used for perioperative optimization in bariatric and orthopedic surgery. Therefore, there may be some applications in other surgical fields, particularly for reducing weight prior to surgery. Rising rates of obesity warrant the evaluation of preoperative GLP-1 agonists in gynecology to reduce surgery morbidity. We reviewed clinical trial-level data on GLP-1 agonist-related weight loss in hopes of completing a cost-effectiveness analysis. However, our review demonstrated that there are no current publications in patients undergoing benign hysterectomy to complete a robust cost-effectiveness analysis. Despite this, this review revealed important information about the use of GLP-1 agonists. It informs both clinicians and patients about realistic expectations for weight loss and how these therapies may be considered in a surgical framework. The goal of this narrative review is to report expected weight loss after these medications, frame the current data into a clinical context in gynecology, and demonstrate the urgent need to scientifically evaluate the role of preoperative GLP-1 agonist use in patients undergoing hysterectomy.

## Introduction and background

By 2030, the most common obesity classification in adult women is expected to be severe obesity (body mass index (BMI) greater than or equal to 40 kg/m^2^), and weight loss management is highly desired [1]. Therefore, glucagon-like peptide 1 (GLP-1) agonists are exceedingly popular. Social media showcases expeditious weight loss with these medications. Contrastingly, in clinical trials, GLP-1 agonists lower weight less dramatically. Regardless, their use could be beneficial as obesity increases surgical morbidity and operative costs (time) in those undergoing benign hysterectomy with BMI over 40 kg/m^2^ [2]. However, it is unclear if this therapy translates to meaningful reductions in BMI, which are related to surgical risk. It is also unknown if delaying surgery to achieve these results is appropriate. Additionally, these medications are expensive. However, short-term, preoperative use would incur less cumulative cost and has been utilized in bariatric surgery. 

Balancing the financial risks of GLP-1 use and risks due to obesity in women’s health surgery is clinically relevant. Therefore, a cost-effective analysis to understand the role of GLP-1 agonist use in hysterectomy-bound patients is warranted. To complete a cost-effectiveness analysis, robust data about GLP-1 agonist-related weight loss are required. Such data are then extrapolated to understand the role of GLP-1 agonists in patients undergoing benign hysterectomy. However, in a quest to understand the relevance of GLP-1 agonists in gynecologic surgery, we determined that there was a paucity of data specific to gynecologic surgery and GLP-1 agonist use and that the weight loss reduction seen in a trial setting for GLP-1 agonists was lower compared to widely publicized outcomes in media. Therefore, more information is needed on GLP-1 agonist use prior to its widespread use in the perioperative space for gynecologic surgery. The goal of this work is to describe weight loss described in rigorous clinical trials, review gynecology-based literature to provide context to these findings, and identify knowledge gaps that require future, dedicated investigation in women’s health. 

## Review

Methods 

**Figure 1:**
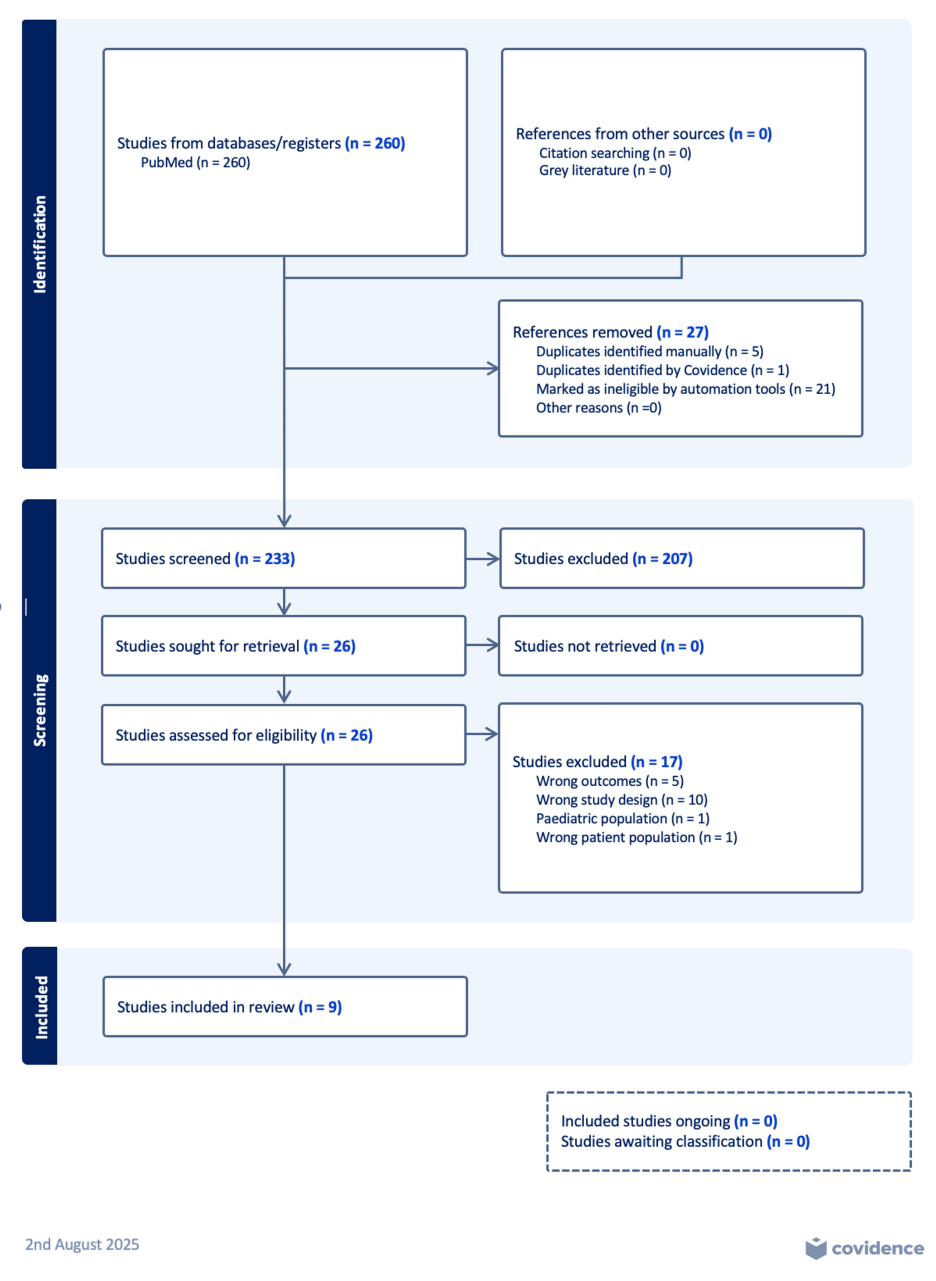
PRISMA diagram of comprehensive literature review of prospective studies and randomized controlled trials on glucagon-like peptide 1 agonist use and weight loss from 2015 to 2025. PRISMA: Preferred Reporting Items for Systematic reviews and Meta-Analysis

A comprehensive literature review using PubMed included randomized controlled trials (RCTs) and prospective studies regarding weight loss in the setting of GLP-1 agonist use from 2015 to 2025. Studies included weight loss data for both sexes. Search terms included English language articles using relevant search terms of the following categories: “glucagon-like peptide 1 receptor agonist”, “exercise”, and “weight loss”. There were 260 articles found on the initial search. Seventeen relevant articles were selected for full-text review. Exclusions include non-clinical study design, retrospective design, and unrelated outcomes. Additional databases were not utilized, such as clinicaltrials.gov, as only published peer-reviewed manuscripts were purposefully selected. Therefore, nine articles were included in the manuscript. The relevant Preferred Reporting Items for Systematic reviews and Meta-Analysis (PRISMA) diagram is seen in Figure 1. Covidence systematic review software (Veritas Health Innovation, Melbourne, Australia) was utilized.

Relevant data were extracted manually by author (A.S.) and reported. Changes in BMI class were calculated based on raw data on weight loss. For these calculations, height was idealized to 1.7 meters (average individual height for both sexes). Risk of bias assessment was not completed as one reviewer completed the review and only RCTs were included. 

An additional secondary search of relevant literature on GLP-1 agonist use, surgery, and gynecology was completed by author (A.S.) to provide context to the results of the comprehensive review. This included focused articles that frame the results of the comprehensive literature search into a clinical context. 

Results

The results of the review are reported in Table 1 [3-11]. The same patient populations were utilized for multiple publications, as denoted in Table 1. The mean study duration ranged from 16 to 68 weeks in the nine included articles [3-11]. Medications included in these articles included semaglutide (SMA) and liraglutide. SMA dosing included intramuscular dosing of 1.7 milligrams (mg)/week or 2.4 mg/week and oral dosing of three, seven, or 14 mg daily. Liraglutide dosing included daily oral use of 1.8 mg or 3 mg. Starting BMI in these studies ranged from 28.2 kg/m^2^ to 39.8 kg/m^2^ [5,9]. Weight loss ranged from 1.1 kg with 26 weeks of daily 3 mg use of SMA to 11.55 kg +/- 0.47 kg with 68 weeks of 2.4 mg weekly use of SMA [6,9]. In 16 weeks, weight loss of 3.4 kg +/- 2.9 kg was reported after 3 mg of daily liraglutide use [8]. In a study of the effect of 3 mg of daily liraglutide use after an eight-week low-calorie diet, all participants underwent the diet program and lost 13.1 kg [7]. The addition of liraglutide for one year (in the absence of rigorous ongoing exercise) after the diet resulted in an additional weight loss of 6.8 kg (total 20.5 kg) [7]. Reduction by one category of BMI class was appreciated in three out of the seven unique patient populations seen in Table 1. 

**Table 1:** Data from the comprehensive review of relevant randomized controlled clinical trials regarding GLP-1 agonist use. BMI: body mass index; kg: kilograms; m: meters; SD: standard deviation; RCT: randomized controlled trial; SMA: semaglutide; GLP-1: Glucagon-like Peptide 1. Asterisk (*) denotes that the same study population was utilized in these publications. SD were reported if present in the original manuscripts.

Year of publication	Author	Type of study	Medication (Dose)	Number of participants	Medication use duration	Mean starting BMI (kg/m^2^) +/- SD	Reported weight loss (kg) +/- SD	Mean completion BMI (kg/m^2^)	Calculation of completion BMI required?	Change in BMI class
2021*	Lundgrenet al. [3]	RCT (eight-week diet program followed by liraglutide)	Liraglutide 3 mg/day	195	52	37.0 +/- 2.9 (Class 2)	13.1 from diet program + 6.8 from liraglutide	32.2 (Class 1)	Yes	Class 2 to Class 1 status
2015	Davies et al. [4]	RCT	Liraglutide 1.8 mg/day	423	56	36.6 (Class 2)	6.4	34.4 (Class 1)	Yes	Class 2 to Class 1 status
			Liraglutide 3 mg/day	211	56	36.6 (Class 2)	2.2	35.8 (Class 2)	Yes	No change
2020	Troneri et al. [5]	RCT	Liraglutide 3 mg/day	282	56	39.3 +/-6.8 (Class 2)	Loss of 6.5% of weight	N/A	N/A	N/A
2022	Kadowaki et al. [6]	RCT	SMA 2.4 mg/week	186	68 weeks	31.6 (Class 1)	11.55 +/- 0.47	27.6 (overweight)	Yes	Class 1 to overweight status
			SMA 1.7 mg/week	93	68 weeks	31.6 (Class 1)	8.53 +/- 0.67	28.6 (overweight)	Yes	
2023*	Jensen et al. [7]	RCT (eight-week diet program followed by liraglutide)	Liraglutide 3 mg/day	195	52 weeks	37.0 +/- 2.9 (Class 2)	13.1 from diet program + 6.8 from liraglutide	32.2 (Class 1)	Yes	Class 2 to Class 1 status
2017	Mensburg et al. [8]	RCT	Liraglutide 3 mg/day	33	16	32.5 +/- 3.7 (Class 1)	3.4 +/- 2.9 kg	31.3 (Class 1)	Yes	No change
2024	Wang et al. [9]	RCT	SMA 3 mg/day	130	26 weeks	28.2 (overweight)	1.1	27.8 (overweight)	Yes	No change
			SMA 7 mg/day	130	26 weeks	28.2 (overweight)	2.2	27.4 (overweight)	Yes	
			SMA 14 mg/day	130	26 weeks	28.2 (overweight)	3.0	27.2 (overweight)	Yes	
2023*	Sandsdal et al. [10]	RCT (eight-week diet program followed by liraglutide)	Liraglutide 3 mg/day	195	52 weeks	37.0 +/- 2.9 (Class 2)	13.1 from diet program + 6.8 from liraglutide	32.2 (Class 1)	Yes	Class 2 to Class 1 status
2015	Pi-Sunyer et al. [11]	RCT	Liraglutide 3 mg/day	2487	56	38.3 +/- 6.4 (Class 2)	8.4 +/- 7.3	35.3 (Class 2)	Yes	No change

As seen by the results of the comprehensive review, the amount of weight loss of GLP-1 agonists alone in a robust clinical trial setting is lower than anticipated from weight loss demonstrated from other media sources. In an observational study of TikTok videos regarding GLP-1 agonist use in 2023, 90% of content creators were not healthcare professionals [12]. Moreover, 82% reported weight loss as the main indication for use of SMA and tirzepatide, which was not Food and Drug Administration indications at the time of the videos, leaving opportunity for misinformation. Although both medications are now approved for chronic weight management, the information regarding weight loss and the degree of it on social and other media may not be accurate. Given that patients are using these avenues to inform healthcare decisions, it is vital for clinicians and surgeons to consider requests for GLP-1 agonist medications in the preoperative setting carefully.

In this comprehensive review, a maximum loss was approximately 26.4 pounds (lbs) after 68 weeks with GLP-1 agonist use alone. After 16 weeks, a weight loss of approximately 14 lbs was seen. When considering this time period, four months is likely a reasonable maximum time to delay surgery for optimization. However, a long delay of surgery may not be appropriate for conditions such as abnormal uterine bleeding or fibroids. In the setting of abnormal uterine bleeding or fibroids requiring surgical intervention, most patients have failed conservative management; therefore, it may be prudent to expedite their care. Given these data, delay of oncologic surgical management for the benefit of GLP-1 agonist-mediated weight loss would not be recommended, as even a delay of eight weeks resulted in adverse outcomes in those awaiting gynecologic oncologic surgery [13]. Similar data regarding delay in surgery and its impact on outcomes in benign gynecology are not available but does raise similar concerns for non-oncologic cases.

Moreover, significant weight loss likely requires both GLP-1 agonist use and lifestyle modifications, which may be difficult in a short preoperative setting. Lastly, GLP-1 agonist treatment decreased weight by one BMI category (Class 1 to overweight class or Class 2 to Class 1 obesity). Given the overall low risk of surgical complications in lower BMI classes during hysterectomies, the impact of these medications may be mixed [2]. In comparing postoperative morbidity in those undergoing abdominal or laparoscopic hysterectomy for benign indications, abdominal hysterectomy posed higher composite morbidity than the laparoscopic approach [14]. The risk of composite morbidity after laparoscopic hysterectomy was noted to be 3%, 4%, and 5% for BMI Class 1, Class 2, and Class 3, respectively [14]. This is compared to 7%, 8%, and 13% for BMI Class 1, Class 2, and Class 3 for abdominal hysterectomy [14]. With greater adoption of laparoscopic hysterectomy, the use of routine abdominal hysterectomy is less common [15]. However, regardless of approach, weight is still a known important modifiable risk factor for surgical morbidity.

In other subspecialities, the use of GLP-1 agonists in the perioperative setting is emerging in hopes of reducing weight before surgery. Current studies are limited. In one single-center, quasi-experimental pilot study of liraglutide for surgical optimization, 37 patients with a mean BMI of greater than or equal to 56.04 kg/m^2^ were given liraglutide for three months [16]. The total weight loss was 5.5% at the three-month mark with a reduction of BMI to 53.05 kg/m^2^ [16]. In a retrospective study of patients undergoing hernia repair, there was no difference in BMI reduction, total weight loss, or 30-day postoperative complications between those who received GLP-1 agonist therapy and those who did not [17]. It is unclear if the described weight reduction causes significant impacts on surgical outcomes, and this has yet to be studied more thoroughly in a robust RCT setting. Although GLP-1 agonist use may be helpful in bariatric patients with higher BMI (50 kg/m^2^ or higher), this patient population and associated BMIs may not be as common in gynecology. Therefore, these results may not be as generalizable to gynecologic patients. 

There are also concerns regarding GLP-1 agonist use. In a retrospective study of 93 patients, there was an increase in intra-abdominal adhesive disease in those on liraglutide 3.0 mg/day for twenty-four weeks preoperatively who underwent laparoscopic sleeve gastrectomy [18]. Moreover, GLP-1 agonist use may increase the risk of sarcopenia, even up to 40%, which may not be suitable given the impact of frailty on postoperative adverse outcomes in the setting of benign hysterectomy [19,20]. GLP-1 agonist use can be extremely expensive for patients. In a cost-effectiveness analysis comparing traditional bariatric surgery and GLP-1 agonist use for obesity management, the monthly cost based on 2023 pricing for both non-compounded SMA and liraglutide was approximately $1600 monthly [21]. Additionally, two months of use is suggested to maintain steady-state and effectiveness of such medications, and finally, within nine months, traditional bariatric surgery was more cost-effective [21]. These nuances are important to consider given the studies on weight loss by GLP-1 agonists utilized these expensive medications, such as SMA and liraglutide, as previously reported in Table 1. In a 2025 cost-effectiveness analysis, bariatric surgery remains the most durable for sustained weight loss given limitations on sustained adherence to GLP-1 agonists [22]. Lastly, in the perioperative period, the use of GLP-1 agonists has also increased the risk of delayed gastric emptying, and clinicians should consider these risks and the subsequent risk of aspiration at the time of surgery when dosing and continuing these medications [23]. Multi-society guidelines have been developed by the American Gastroenterological Association, American Society for Metabolic and Bariatric Surgery, American Society of Anesthesiologists, International Society of Perioperative Care of Patients with Obesity, and Society of American Gastrointestinal and Endoscopic Surgeons to address these concerns [23]. Specific guidelines from the American College of Obstetrics and Gynecology have not been developed for GLP-1 agonist use in the gynecologic setting. 

Strengths of this manuscript include a thorough review of RCT data regarding GLP-1 agonist use and weight loss. An additional inclusion of relevant and focused articles on surgery and gynecology was also completed to provide context for the findings of the comprehensive review. This was done purposefully as there are no current data on GLP-1 agonist use in gynecology, and there are very limited data on GLP-1 agonist use and surgery in other fields. Including both searches provides a clearer clinical picture of the utility of GLP-1 agonist use in the perioperative setting. This manuscript also answers an important question about the expected weight loss for patients on GLP-1 agonist therapy and frames its use within the perioperative context. 

A significant limitation is the inclusion of articles in non-perioperative settings. We were only able to comment on weight loss expected after GLP-1 agonist use in the medical setting as there were no robust RCT-level data in the surgical setting. Additionally, we were unable to evaluate non-weight-based benefits of GLP-1 agonist use. Patients who are using GLP-1 agonists may be inherently more motivated to lose weight and may improve their lifestyles and overall health. The included studies did not report undesirable side effects of GLP-1 agonist use in the perioperative setting. For example, side effects reported in the non-operative setting, such as nausea, constipation, and pancreatitis, may have significant clinical impacts in the postoperative period. We were unable to review the impact of GLP-1 agonists on other comorbid conditions that may impact surgical risk, such as obstructive sleep apnea or diabetes. Additionally, risk of bias analysis was not completed, given one reviewer completed the review; however, only RCT articles were included, which may lower bias risk due to study design. However, this review is timely given the recent interest in GLP-1 agonist use, yet there are limited prospective data to inform both surgeons and patients in gynecology.

## Conclusions

In conclusion, the preoperative GLP-1 agonist use should be actively studied in the setting of hysterectomies in benign gynecology, especially with rising rates of obesity in women. By understanding the data surrounding GLP-1 agonist use, surgeons can more effectively inform patients, plan the timing of surgical interventions, and understand the expected weight loss after such medications. This can also be helpful when considering the overall cost of these medications and the possible risk of side effects that may impact surgical care. Surgeons considering preoperative GLP-1 agonist use should inform patients of the risks of possible financial toxicity, side effects, and surgical delay for weight loss. However, it is important to consider that preoperative use of these medications could be beneficial and that information is simply unknown in the current literature in gynecology. Although there are limited data on this topic, this manuscript frames our understanding of the weight loss caused by preoperative GLP-1 agonists and calls for targeted future investigations in gynecology prior to broad implementation of GLP-1 agonists in the perioperative setting. 

## Appendices

Topic: comprehensive search criteria for comprehensive review of weight loss after GLP-1 agonist use 

Concept: GLP-1 Agonists

"Glucagon-Like Peptide-1 Receptor Agonists"[Mesh] OR "Glucagon-Like Peptide-1 Receptor Agonists" [Pharmacological Action] OR "Semaglutide"[Supplementary Concept] OR "Liraglutide"[MeSH] OR "Exenatide"[MeSH] OR "Tirzepatide"[MeSH] OR "Glucagon-Like Peptide-1 Receptor Agonist*"[tiab] OR "GLP-1 agonist*"[tiab] OR "GLP1 agonist*"[tiab] OR semaglutide[tiab] OR liraglutide[tiab] OR exenatide[tiab] OR tirzepatide[tiab] OR Ozempic[tiab] OR wegovy[tiab] OR saxenda[tiab] OR Victoza[tiab] OR mounjaro[tiab] OR zepbound[tiab]

Concept: Exercise

"Exercise"[MeSH] OR exercise[tiab] OR exercises[tiab] OR exercising[tiab] OR walking[tiab] OR jogging[tiab] OR running[tiab] OR "strength training"[tiab] OR swimming[tiab] OR "resistance training"[tiab] OR "high-intensity interval training"[tiab]

Concept: Weight Loss

"Weight Loss"[MeSH:NoExp] OR "weight loss"[tiab] OR "lost weight"[tiab] OR "losing weight"[tiab] OR "weight reduction"[tiab] OR "weight reductions"[tiab] OR "reducing weight"[tiab]

Full Search to Copy/Paste into PubMed

((("Glucagon-Like Peptide-1 Receptor Agonists"[Mesh] OR "Glucagon-Like Peptide-1 Receptor Agonists" [Pharmacological Action] OR "Semaglutide"[Supplementary Concept] OR "Liraglutide"[MeSH] OR "Exenatide"[MeSH] OR "Tirzepatide"[MeSH] OR "Glucagon-Like Peptide-1 Receptor Agonist*"[tiab] OR "GLP-1 agonist*"[tiab] OR "GLP1 agonist*"[tiab] OR semaglutide[tiab] OR liraglutide[tiab] OR exenatide[tiab] OR tirzepatide[tiab] OR Ozempic[tiab] OR wegovy[tiab] OR saxenda[tiab] OR Victoza[tiab] OR mounjaro[tiab] OR zepbound[tiab]) AND ("Exercise"[MeSH] OR exercise[tiab] OR exercises[tiab] OR exercising[tiab] OR walking[tiab] OR jogging[tiab] OR running[tiab] OR "strength training"[tiab] OR swimming[tiab] OR "resistance training"[tiab] OR "high-intensity interval training"[tiab])) AND ("Weight Loss"[MeSH:NoExp] OR "weight loss"[tiab] OR "lost weight"[tiab] OR "losing weight"[tiab] OR "weight reduction"[tiab] OR "weight reductions"[tiab] OR "reducing weight"[tiab]) AND English[lang]) NOT ((Editorial[pt] OR Letter[pt] OR Case Reports[pt] OR Comment[pt] OR Congress[pt])). 
